# Radiotherapy for asymptomatic brain metastasis in epidermal growth factor receptor mutant non-small cell lung cancer without prior tyrosine kinase inhibitors treatment: a retrospective clinical study

**DOI:** 10.1186/s13014-015-0421-9

**Published:** 2015-05-27

**Authors:** SongRan Liu, Bo Qiu, LiKun Chen, Fang Wang, Ying Liang, PeiQiang Cai, Li Zhang, ZhaoLin Chen, ShiLiang Liu, MengZhong Liu, Hui Liu

**Affiliations:** State Key Laboratory of Oncology in South China, Collaborative Innovation Center for Cancer Medicine, Guangzhou, 510060 China; Guangdong Esophogeal Cancer Research Institute, Guangzhou, China; Department of Radiation Oncology, Sun Yat-sen University Cancer Center, State Key Laboratory of Oncology in South China; Collaborative Innovation Center for Cancer Medicine, Guangzhou, 510060 China; Department of Medical Oncology, Sun Yat-sen University Cancer Center, Guangzhou, China; Department of Molecular Diagnosis, Sun Yat-sen University Cancer Center, Guangzhou, China; Department of Radiology, Sun Yat-sen University Cancer Center, Guangzhou, China

**Keywords:** Asymptomatic brain metastasis, Radiotherapy, Chemotherapy, Epidermal growth factor receptor mutation, Tyrosine kinase inhibitor

## Abstract

**Background:**

Non-small cell lung cancer (NSCLC) with brain metastasis (BM) harboring an epidermal growth factor receptor (EGFR) mutation shows good response to tyrosine kinase inhibitors (TKIs). This study is to assess the appropriate timing of brain radiotherapy (RT) for asymptomatic BM in EGFR mutant NSCLC patients.

**Methods:**

There were 628 patients diagnosed with EGFR mutant NSCLC between October 2005 and December 2011. Treatment outcomes had been retrospectively evaluated in 96 patients with asymptomatic BM without prior TKI treatment. 39 patients received first-line brain RT, 23 patients received delayed brain RT, and 34 patients did not receive brain RT.

**Results:**

With a median follow-up of 26 months, the 2-year OS was 40.6 %. Univariate analyses revealed that ECOG performance status (p = 0.006), other distant metastases (p = 0.002) and first line systemic treatment (p = 0.032) were significantly associated with overall survival (OS). Multivariate analyses revealed that other sites of distant metastases (p = 0.030) were prognostic factor. The timing of brain RT was not significantly related to OS (p = 0.246). The 2-year BM progression-free survival (PFS) was 26.9 %. Brain RT as first-line therapy failed to demonstrate a significant association with BM PFS (p = 0.643).

**Conclusions:**

First-line brain RT failed to improve long-term survival in TKI-naïve EGFR mutant NSCLC patients with asymptomatic BM. Prospective studies are needed to validate these clinical findings.

## Background

Brain metastasis (BM) is a common complication of lung cancer and is associated with poor treatment outcomes. BM is observed in approximately 25–30 % of non-small cell lung cancer (NSCLC) patients [1]. The median survival is approximately 4–11 weeks in untreated patients but can be improved by whole-brain radiation therapy (WBRT) to 3–6 months [2]. However, NSCLC has been regarded as a relatively radio-resistant malignancy, and 30 Gy WBRT may be insufficient to destroy the lesions; recent studies have suggested that the median response rate to WBRT remains approximately 25–30 % [3]. The role of chemotherapy in the treatment of brain metastasis remains controversial.

Advances in the understanding of the molecular biology of tumors have led to the development of targeted agents with promising results in the treatment of NSCLC. Epidermal growth factor receptor (EGFR) mutations are associated with a significant sensitivity to EGFR tyrosine kinase inhibitors (TKI), which can significantly improve treatment outcome [4]. Recently, the efficacy of epidermal growth factor receptor tyrosine kinase inhibitors (EGFR-TKIs) for NSCLC patients with BM has been reported [5, 6]. Moreover, several reports demonstrate that NSCLC patients with mutant EGFR and BM could also achieve favorable outcomes when treated with EGFR-TKIs as single-agent chemotherapy. Several studies have reported that TKI treatment results in high response rates (70–89 %) and increased overall survival (OS) and progression-free survival (PFS) (12.9–19.8 months and 6.6–23.3 months, respectively) in selected populations of EGFR-mutated NSCLC patients with BM [7–9].

Several studies have suggested that patients with BM harboring EGFR mutations may have higher response rates to WBRT than those with wild-type tumors [10–13]. However, unlike the EGFR-mutant primary lung tumor, 11–44 % of brain metastases exhibit resistance to TKI treatment [7, 8]. In addition, Omuro et al. [14] reported a high incidence of central nervous system (CNS) metastases during the course of a standard treatment of gefitinib, an EGFR inhibitor, despite good control of other disease sites. These results suggest that local therapy may still be important for the treatment of BM in patients with EGFR mutations.

However, for EGFR-mutant NSCLC patients with asymptomatic brain metastasis who do not require urgent symptom relief, the proper treatment schedule is not well established. Therefore, we sought to gain insight from the retrospective analysis of patients treated with different combinations of irradiation/TKI therapies.

## Methods

### Acquisition of clinical data

A total of 628 patients were diagnosed with adenocarcinoma of the lung harboring EGFR mutations between October 2005 and December 2011 at the Sun Yat-Sen University Cancer Center. Treatment outcomes had been retrospectively evaluated in 96 patients with asymptomatic BM without prior TKI treatment. Before receiving treatment, each patient underwent a physical examination, laboratory tests and electrocardiograms as well as a medical history evaluation, including documentation of concomitant medications, performance status, and smoking history. Patient data included chest and upper abdomen computed tomography (CT) scans or positron emission tomography (PET) scans, bone scans, and magnetic resonance imaging (MRI) of the brain. Tumor stage was classified using the tumor/node/metastasis (TNM) system proposed by the American Joint Committee on Cancer (8th edition). T and N stage were determined on the basis of the findings of CT with or without additional fiberoptic bronchoscopy. Mediastinal lymph nodes ≥1 cm on transaxial CT images or SUV ≥ 2.5 on PET scans were considered positive. All patients were required to meet the following inclusion criteria: 1) pathologically confirmed NSCLC harboring an activating EGFR mutation; 2) documented measurable brain metastases (AJCC stage IV disease) at first diagnosis; and 3) Eastern Cooperative Oncology Group (ECOG) performance status ≤3. Patients with severe comorbid conditions and other active malignancies were excluded from the analysis.

### EGFR Genotyping

All patients provided written informed consent for the comprehensive use of molecular analysis. Genomic DNA was isolated from paraffin-embedded tissues, which were obtained by transbronchial lung biopsy or from cytologic materials, such as bronchial lavage fluid and pleural effusions. Methanol-fixed cytologic specimens were used for DNA extraction for patients for whom only cytologic sample were available at initial diagnosis. Epidermal growth factor receptor mutation analysis of all of the patients was performed by direct sequencing or the ARMS method at the Department of Molecular Diagnosis [15].

### Follow-up and treatment response assessment

The beginning of the follow-up period was defined as the initial date of local or systemic treatment. Patients underwent chest, abdomen, and pelvic CT and brain MRI every three months until disease progression. Bone scans were administered when patients were suspected of having bone metastasis. Positron emission tomography scans were administered when systemic progression was expected. The rates and times of treatment response, overall survival, local relapse and distant metastases were recorded.

Systemic disease at the time of BM diagnosis was considered active if a chest, abdomen and pelvic CT, PET, and/or bone scan performed within four weeks of the BM diagnosis revealed new sites of extra-cranial metastases or progression at previously known sites of disease. The time to brain progression was measured from the initial date of local or systemic treatment until the date of radiological progression or worsening neurological symptoms at the time of last follow-up.

### Statistical analysis

The study endpoint was OS. Overall survival was calculated as the time from the initial date of local or systemic treatment to the date of death from any cause or to the last visit before March 31, 2014, censored at the date of last follow up. Each variable was assessed first in univariate analysis, and the variables with a P value <0.10 were evaluated by multivariate analysis. Survival curves were plotted using the Kaplan-Meier method. We fit the proportional hazards model using Cox regression. After testing for variable interactions, a forward stepwise elimination procedure was used to determine the best-fitting model. P values <0.05 were regarded as statistically significant in multivariate analysis. All statistical analyses were performed using SPSS 19.0 software (IBM).

### Ethics statement

Participants’ information collection was guaranteed by Ethics Committee of Sun Yat-sen University Cancer Center. Written informed consent was not obtained, instead, all clinical records was anonymized and de-identified prior to analysis. All authors have read and approved the manuscript.

## Results

### Patient characteristics

The patient characteristics are detailed in Table 1. The study included 96 patients, with 62 females and 34 males. There were 50 patients with BM at the initial diagnosis of NSCLC (stage IV), the other 46 patients developed BM after first-line treatment. None of the patients received TKI therapy before the diagnosis of BM. None of the patients experienced any symptoms related to the metastatic brain tumors. After diagnosis and general evaluation, all 96 patients received either chemotherapy or a TKI as systemic treatment. The regimens included cisplatin or carboplatin combined with paclitaxel or pemetrexed. All 61 patients exhibited disease progression and were administered a TKI as second-line systemic treatment after the diagnosis of BM. The 35 patients who received TKI as first-line therapy after the diagnosis of BM were treated with 250 mg of oral gefitinib or 150 mg of erlotinib once daily until disease progression or unacceptable toxicity.

**Table 1:** Clinical and molecular characteristics of patients (n = 96)

Charateristics	Patients (n = 96) No.(%)
Sex	
Male	34 (35.4 %)
Female	62 (64.6 %)
Age(year), median(range)	54 (26–79)
^a^ECOG performance status	
0–1	69 (71.9 %)
2–3	27 (28.1 %)
No. of brain lesions	
1–4	32 (33.3 %)
>4	64 (66.7 %)
Max size of brain lesions	
<1 cm	75 (78.1 %)
>1 cm	21 (21.9 %)
Location of brain lesions	
Brain stem mets	13 (13.5 %)
Non-brain stem mets	83 (86.5 %)
Other sites of metastatic disease	
Yes	61 (63.5 %)
No	35 (36.5 %)
^b^EGFR mutation	
Exon 19	45 (46.9 %)
Exon 21	51 (53.1 %)
First line systemic treatment	
Chemotherapy	61 (63.5 %)
^c^TKI	35 (36.5 %)
Time of Brain radiotherapy	
First line	39 (40.6 %)
Delayed	23 (24.0 %)
No ^d^RT	34 (35.4 %)

^a^Eastern Cooperative Oncology Group

^b^Epidermal growth factor receptor

^c^Tyrosine kinase inhibitors

^d^Radiotherapy

### Survival and prognostic factors

**Fig. 1:**
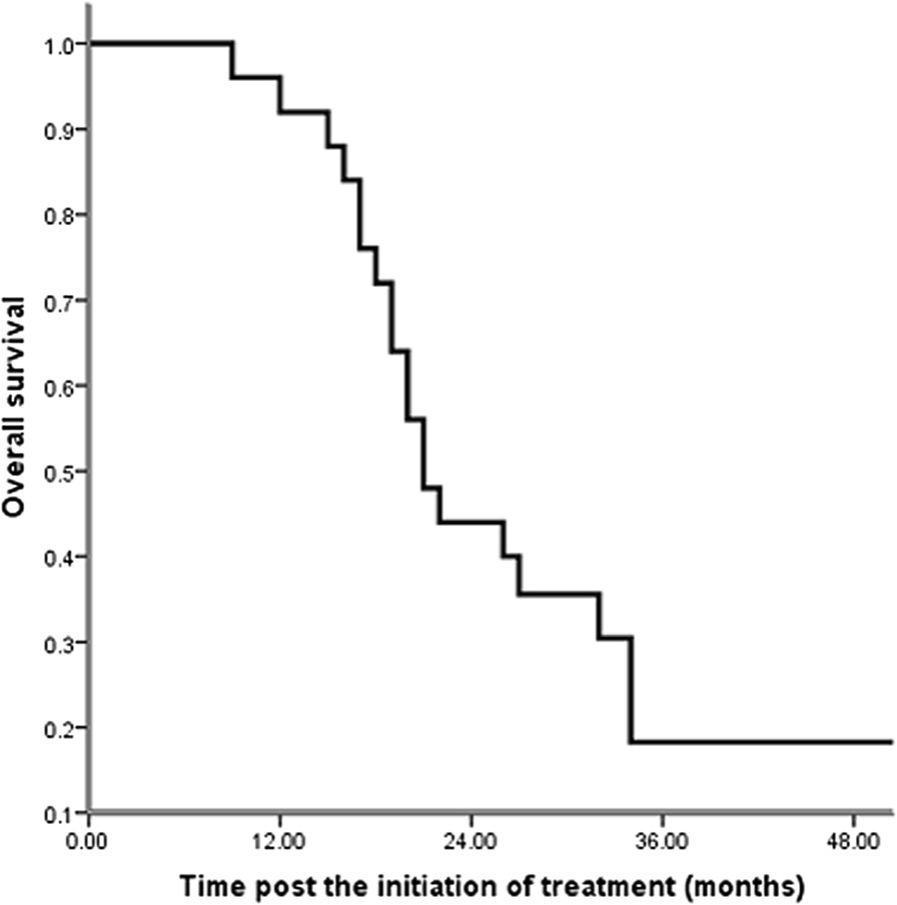
Overall survival since the initiation of treatment (n = 96): The 2-year OS was 40.6 %, with a median follow-up of 26 months (range, 11–56 months), and the estimated overall survival time was 21.0 months

With a median follow-up of 26 months (range, 11–56 months), the 2-year OS was 40.6 %, and the estimated OS time was 21.0 months (Fig. 1). Eastern Cooperative Oncology Group performance status (p = 0.006), other distant metastases (p = 0.002) and first line systemic treatment (p = 0.032) were significantly associated with OS (Table 2). The clinical factors that were statistically significant (p < 0.10) in a univariate analysis were analyzed further in a multivariate analysis with a stepwise selection of variables. Only other sites of distant metastases (p = 0.030) were selected by a stepwise selection of factors in the final models (Table 3).

**Table 2:** Univariate analysis of prognostic factors of overall survival (n = 96)

Variable	HR, 95 % CI	p value
Sex	0.81 (0.43–1.55)	0.534
(male vs. female)		
Age	0.74 (0.39–1.41)	0.342
(>54 years vs. ≤54 years)		
ECOG performance status	3.24 (1.40–7.50)	0.006
(0–1 vs. 2–3)		
No. of brain metastases	1.41 (0.74–2.69)	0.299
(1–4 vs. >4)		
Max size of brain lesions	1.03 (0.67–1.76)	0.763
(<1 cm vs. >1 cm)		
Location of brain lesions	1.62 (0.98–2.73)	0.267
(Brain stem mets vs. Non–brain stem mets)		
Other sites of metastatic disease	3.89 (1.58–9.57)	0.002
(No vs. Yes)		
EGFR mutation	0.74 (0.39–1.40)	0.351
(Exon 19 vs. Exon 21)		
First line systemic treatment	2.43 (1.08–5.48)	0.032
(TKI vs. Chemotherapy)		
Time of brain RT	0.77 (0.50–1.19)	0.246
(First line vs. Delayed vs. No RT)		

Legend: Each variable was assessed first in univariate analysis, with P value <0.10 regarded as statistically significant. Instead of timing of brain radiotherapy (RT), only Eastern Cooperative Oncology Group (ECOG) performance status, other distant metastases and first line systemic treatment were significantly associated with the overall survival

**Table 3:** Multivariate analysis of prognostic factors for overall survival (n = 96)

Variable	HR, 95 % CI	p value
Other distant metastases	3.53 (1.13–11.08)	0.030
(No vs. Yes)		

Legend: The clinical factors that were statistically significant (p < 0.10) in a univariate analysis were analyzed further in a multivariate analysis with a stepwise selection of variables. P values <0.05 were regarded as statistically significant in multivariate analysis. Only other sites of distant metastases were selected by a stepwise selection of factors in the final models

### Difference in outcomes by brain RT

**Fig. 2:**
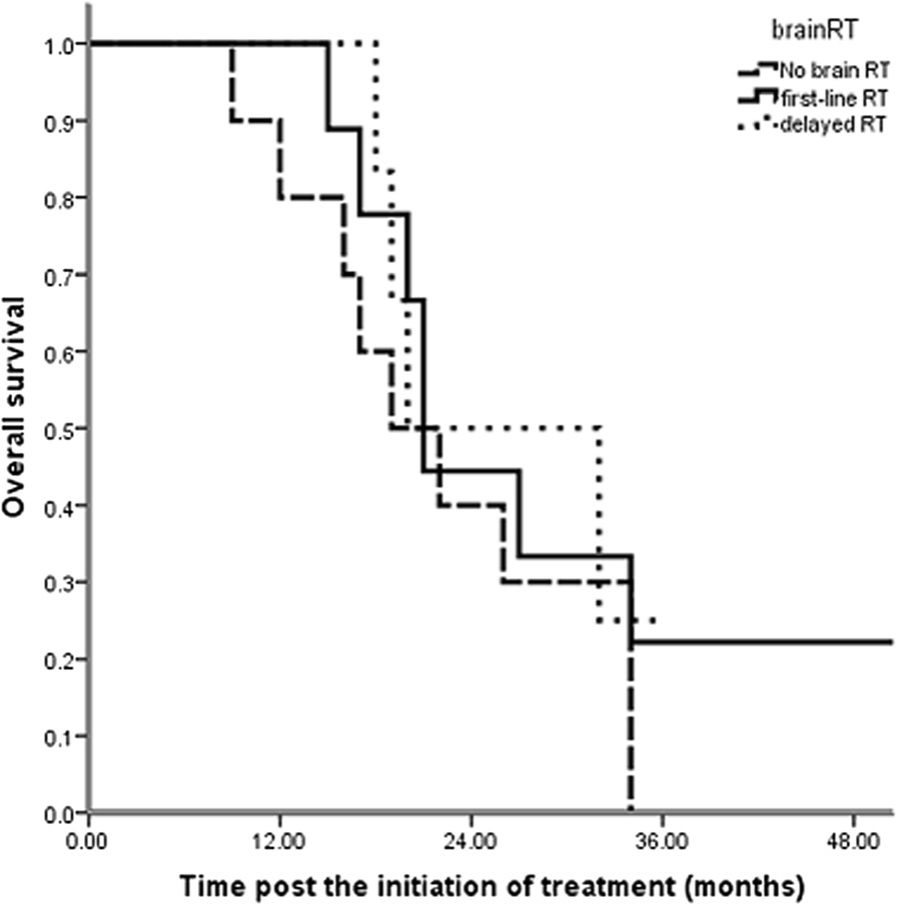
Impact of the timing of brain RT on overall survival (p = 0.246, n = 96): 39 patients were treated initially with brain radiotherapy (first-line RT), and 23 patients were administered RT when they exhibited brain disease progression (delayed RT). Thirty-four patients did not receive radiotherapy and remained asymptomatic until the last follow-up (no brain RT). Univariate analysis revealed that the timing of brain RT was not significantly related to OS (p = 0.246)

**Fig. 3:**
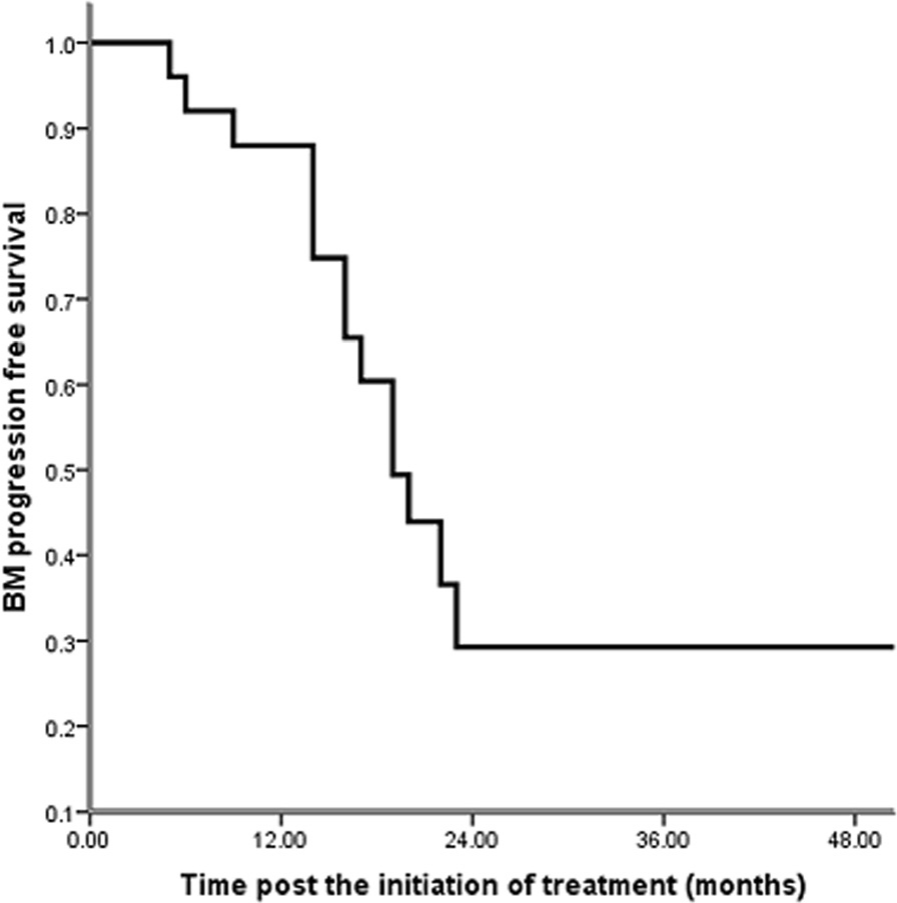
Brain metastasis progression free survival since the initiation of treatment (n = 96): 54 patients exhibited progressive brain disease after local or systemic treatment. The 2-year brain metastasis progression free survival (BM PFS) was 29.6 %, and the estimated BM PFS time was 17 months

**Fig. 4:**
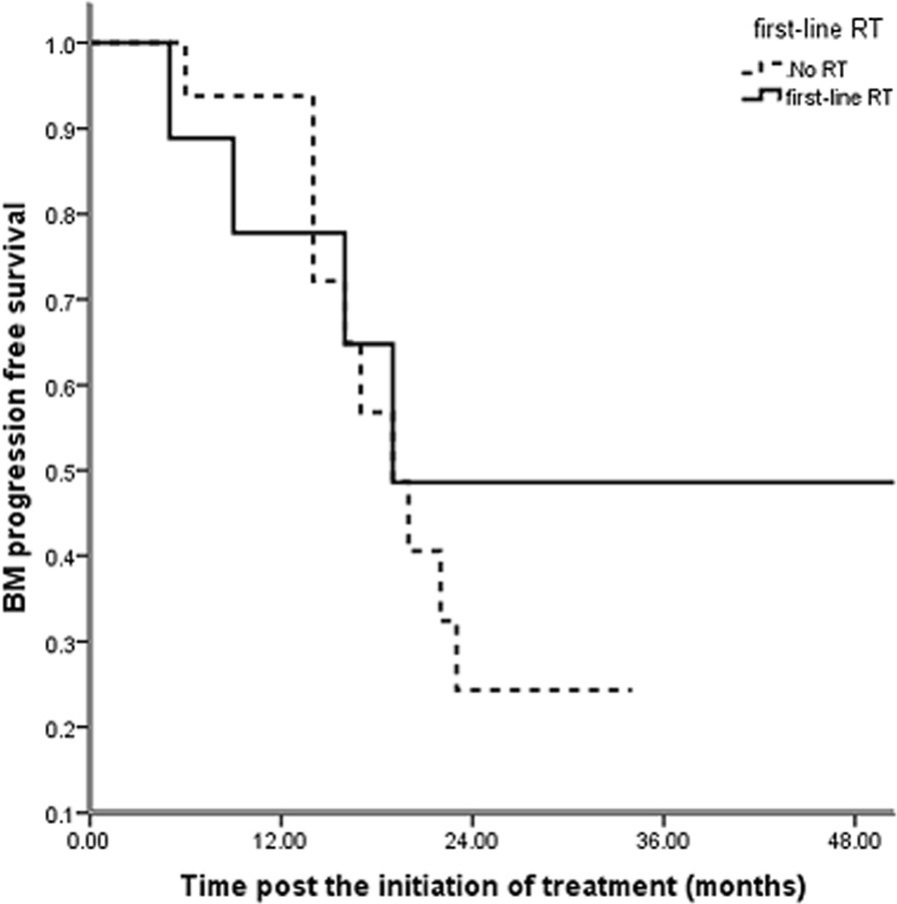
Impact of the timing of brain RT on BM PFS (p = 0.643, n = 96): Brain RT as first-line therapy was not significantly associated with BM PFS (p = 0.643, HR 0.82, 95 % CI 0.39–1.81)

Thirty-nine patients were treated initially with brain RT (13 with stereotactic radiosurgery and 26 with WBRT), and 23 patients were administered RT when they exhibited brain disease progression (eight with stereotactic radiosurgery and 15 with WBRT). Thirty-four patients did not receive radiotherapy and remained asymptomatic until the last follow-up. Univariate analysis revealed that the timing of brain RT was not significantly related to OS (p = 0.246, Table 2, Fig. 2). During the follow-up, 54 patients exhibited progressive brain disease after local or systemic treatment. The 2-year BM PFS was 29.6 %, and the estimated BM PFS time was 17 months (Fig. 3). Brain RT as first-line therapy was not significantly associated with BM progression-free survival (p = 0.643, HR 0.82, 95 % CI 0.39-1.81, Fig. 4).

**Fig. 5:**
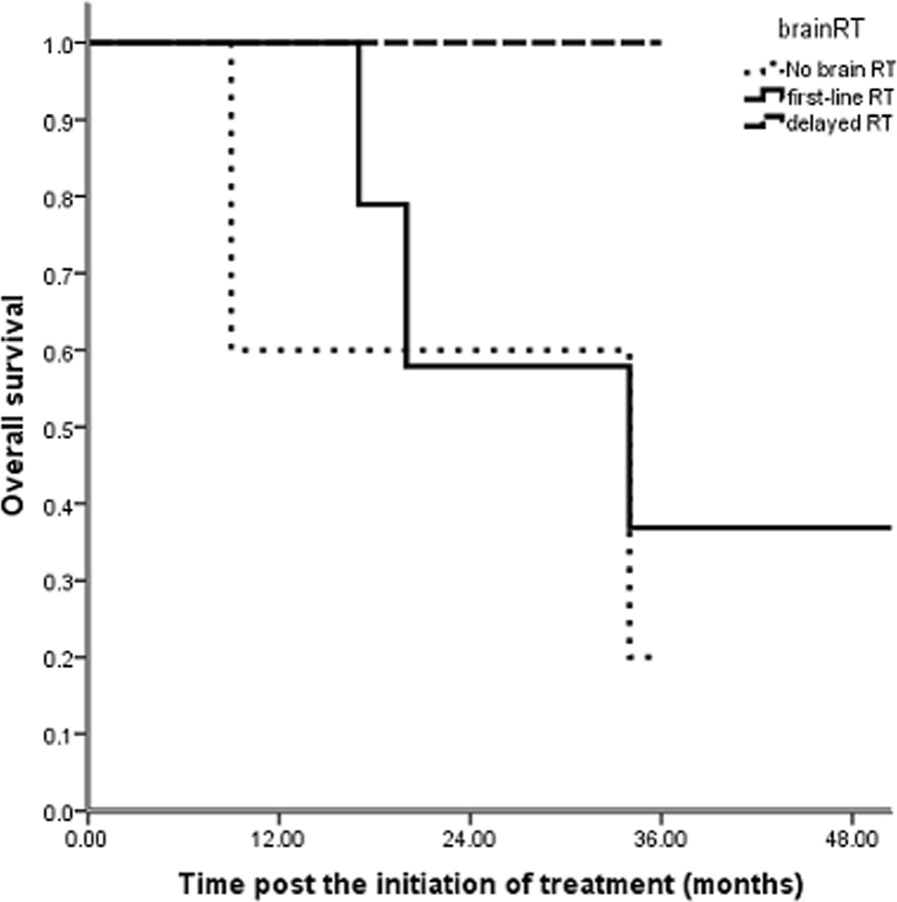
Impact of the timing of brain RT on brain-metastases-only patients’ overall survival (p = 0.019, n = 35): The timing of brain RT was significantly related to OS (P = 0.019). Patients with delayed brain RT had better long-term survival than the others

There were 35 patients with brain metastases only, 19 patients received immediate brain RT, eight patients had delayed brain RT, and eight patients did not receive brain RT. Univariate analysis showed that the timing of brain RT was significantly related to OS (P = 0.019, Fig. 5). Patients with delayed brain RT had better long-term survival than the others.

### Pattern of treatment failure

Of the 72 (72/96, 75 %) patients who died during follow-up, 65 (65/96, 68 %) patients died of disease progression, and the remaining seven died of unknown reasons. Among the 65 patients, 46 (46/65, 71 %) patients exhibited extra-cranial lesion progression, seven (7/65, 11 %) patients exhibited intra-cranial lesion progression only, and 12 (12/65, 18 %) patients exhibited both intra- and extra-cranial lesion progression. Patients received crossover therapies (TKI-Chemotherapy or Chemotherapy-TKI) when they exhibited systemic disease progression.

### Toxicities

The most frequent toxicities observed were nausea, vomiting, neutropenia and skin rash, and the majority of toxicities were Grade 2 (G2). Grade 3 vomiting was observed in 24 out of 96 patients (25 %). Grade 3 neutropenia occurred in 23 out of 96 patients (24 %). Grade 3 skin rash was observed in 4 out of 96 patients (4.0 %). No G3-5 neurotoxicity was reported for patients who received brain RT. No treatment-related deaths were recorded.

## Discussion

This study retrospectively analyzed a consecutive cohort of NSCLC patients tested for EGFR mutations. Our results suggest that among the EGFR-mutated patients with asymptomatic BMs, the timing of brain RT was not significantly related to OS (p = 0.246). Furthermore, subgroup analysis revealed that brain RT as first-line therapy was not significantly associated with BM progression-free survival (p = 0.643). Only the status of other sites of distant metastases strongly influenced survival after the commencement of treatment (p = 0.030).

Whole brain radiotherapy is generally the standard treatment in patients with multiple brain metastases, as it addresses both macroscopic and microscopic disease. Whole brain radiotherapy was shown to result in an improvement in symptoms in 64–85 % of patients [16–18] and in a prolonged median survival of 3–6 months [2]. However, WBRT exhibits both short- and long-term toxicity. Patients may experience continued fatigue, nausea, neurocognitive deficits and other general or focal neurologic symptoms during and after the treatment [19, 20]. Soffietti et al. reported the health-related quality-of-life (HRQOL) results of adjuvant WBRT compared with observation after either surgery or radiosurgery for a limited number of brain metastases in patients with stable solid tumors. Overall, patients in the observation arm reported better HRQOL scores than those who received WBRT. The differences were clinically relevant during the early follow-up period (for global health status at 9 months, physical functioning at 8 weeks, cognitive functioning at 12 months, and fatigue at 8 weeks) [21].

The prevalence of EGFR mutations in BM from NSCLC patients remains to be determined. Matsumoto et al. [22] and Gow et al. [11] have reported EGFR mutations in 63 % and 44 % of BM in an Asian study cohort, respectively. This prevalence is similar to that reported in primary lung lesions, varying from 30 % to 50 % [23, 24]. Eichler et al. [10] reported that in a cohort of 93 NSCLC patients with BM, 41 patients (44 %) had mutations in EGFR. The study demonstrated that a large portion of NSCLC patients with BMs exhibited EGFR mutations. Solitary BMs in the absence of other distant metastases were more common in NSCLC patients with wild-type EGFR compared with patients with mutated EGFR (31 vs. 7 %, and 35 vs. 12 %, respectively). In the study by Eichler et al., 18 % of patients with BM were treated initially with WBRT either alone (53 %) or in combination with craniotomy for neurosurgical resection (22 %) or stereotactic radiosurgery (8 %). The median survival from the time of BM was 11.7 months and was longer for patients with an EGFR mutation (14.5 vs. 7.6 months, p = 0.09). Multivariable analysis suggested that an EGFR mutation (HR: 0.50, 95 % CI: 0.30–0.82), age (HR: 1.03, 95 % CI: 1.00–1.05), and active extra-cranial disease (HR: 3.30, 95 % CI: 1.70–6.41) were independently associated with survival. Gow et al. [11] retrospectively analyzed 63 NSCLC patients with BM treated by WBRT and found that 46 patients carrying EGFR mutations exhibited a median survival of 17.3 months compared with 6.6 months for patients with wild-type EGFR. Additionally, 54 % of patients with EGFR mutations responded to WBRT compared with only 24 % in the wild-type group.

Recent studies have reported that despite the issue of the blood brain barrier, patients with EGFR-mutated BM exhibit positive responses to TKIs in addition to the promising response rate to brain RT. Preclinical studies in mouse models of EGFR-mutant NSCLC BM have demonstrated the treatment efficacy of gefitinib. [25] A recent phase II study with 28 patients [26] prospectively evaluated the efficacy of EGFR TKIs in the treatment of metastatic brain tumors in NSCLC patients harboring EGFR mutations. Twenty-three patients (83 %) exhibited a partial response (PR), and 3 patients (11 %) had stable disease (SD), representing a disease control rate of 93 %. The median PFS and OS were 6.6 months (95 % CI, 3.8–9.3 months) and 15.9 months (95 % CI, 7.2–24.6 months), respectively. There was no difference in PFS and OS with respect to the EGFR TKI used. After discontinuation of the treatment, 14 patients (50 %) received local therapy (whole-brain radiotherapy or radiosurgery) for BM during their disease course, with a local therapy-free interval of 12.6 months (95 % CI, 7.6–17.6 months). The results implied that EGFR TKI therapy may represent the treatment of choice for BMs in NSCLC patients harboring an activating EGFR mutation. Gerber et al. also reported the results of erlotinib versus radiation therapy for brain metastases in 110 patients with EGFR-mutant lung adenocarcinoma and found that there was no significant difference in OS between the WBRT and erlotinib groups (median, 35 vs. 26 months; P = 0.62) [27].

In the current study, 23 (23/96, 24 %) patients received delayed brain RT, because the brain tumors were stable radiologically with no obvious neurologic symptoms during initial systemic treatment. Among these 23 patients, 13 (13/23, 56.5 %) exhibited responses to brain RT, 6 (6/23, 26.1 %) had stable disease, and 4 (4/23, 17.4 %) developed intra-cranial disease progression. Four (4/23, 17.4 %) patients died of intra-cranial disease progression, and 11 (11/23, 47.8 %) patients died of active distant metastases in other sites. As not all patients received brain RT during the disease course and given the long-term neurotoxicity of WBRT and stereotactic radiosurgery, a better treatment option may be upfront treatment with EGFR TKIs followed by brain RT at the appropriate time when neurologic symptoms or signs develop. There were 35 patients with brain metastases only in current study, 19 patients received immediate brain RT, 8 patients had delayed brain RT, and 8 patients did not receive brain RT. Patients in this subgroup were more likely to have aggressive treatment. It might because patients with brain metastasis only were believed to have better treatment outcome than those with multiple sites metastases. However, univariate analysis showed that patients with delayed brain RT had better long term survival. This result suggested that sequential treatment (TKI followed by brain RT when brain lesions progress) might be an optimal choice for these patients. Since the number of patients in this subgroup remained limited, further studies should be conducted on this finding in the future.

Tumors with exon 19 deletions exhibited a higher incidence of CNS involvement compared with tumors bearing the L858R mutation (21 vs. 3 %). Sekine et al. [28] reported that compared with the wild-type EGFR group and the exon 21 point mutation group, NSCLC patients with the exon 19 deletion exhibit a peculiar pattern of brain metastases, including multiple small metastases with little brain edema. This metastatic pattern may be similar to that of miliary brain metastases. In our study, we included 45 patients with the exon 19 deletion and 51 patients with the exon 21 point mutation. Among the 64 patients with multiple (more than 4) BM lesions, 36 patients exhibited the exon 19 deletion, and 28 patients had the exon 21 point mutation. There was no significant difference between the two EGFR mutation types with respect to the numbers of BMs (p = 0.934). Considering all 96 patients, we failed to observe any significant differences in OS (p = 0.362, HR 0.75, 95 % CI 0.39–1.41) between the two types of EGFR mutations. The mechanism explaining the difference in intra-cranial spread remains unknown. Epidermal growth factor receptor mutations with the exon 19 deletion have been proposed to reduce the growth capacity of tumor cells, leading to smaller-sized BMs. In our study, we did not observe this phenomenon, possibly because only patients free of CNS symptoms were enrolled in the analysis.

There are several potential limitations of our current study. First, patients enrolled in the study received treatment between October 2005 and December 2011; therefore, 61 patients were administered chemotherapy as first-line systemic treatment, and only 35 patients received TKIs as first-line treatment. Most of the patients received crossover regimens (chemotherapy-TKI or TKI-chemotherapy). Second, although recent studies reported a discordance rate of 27–28 % for the EGFR mutation status between the primary and metastatic sites [29, 30], pathological and genetic confirmation of BM could not be performed. Third, there were several other limitations, including various chemotherapy regimens and the short follow-up time. Fourth, there may have been a selection bias with respect to patients requiring upfront RT and those patients in whom RT could be delayed. It is possible that the patients with deferred RT did well because they had a lower burden of CNS disease and did not require early WBRT.

## Conclusions

In conclusion, this study revealed that EGFR TKIs are very effective for treating NSCLC patients harboring EGFR mutations and asymptomatic brain metastases. First-line brain RT did not improve long-term survival in this cohort of patients. The status of systemic disease was the strongest prognostic factor for stage IV EGFR-mutated NSCLC patients. In TKI-naïve patients with an EGFR mutation and asymptomatic BM, it may be proper to initiate EGFR TKI therapy and defer upfront brain RT, particularly in those with active systemic disease. Prospective studies are needed to validate these clinical findings.

## Abbreviations

BM: Brain metastasis
CI: Confidence interval
CNS: Central nervous system
CT: Computed tomography
ECOG: Eastern Cooperative Oncology Group
EGFR: Epidermal Growth Factor Receptor
EGFR-TKIs: Epidermal growth factor receptor tyrosine kinase inhibitors
HR: Hazard ratio
MRI: Magnetic resonance imaging
NSCLC: Non-Small Cell Lung Cancer
OS: Overall survival
PET: Positron emission tomography
PFS: Progression-free survival
PR: Partial response
RT: Radiotherapy
SD: Stable disease
TKI: Tyrosine kinase inhibitors
TNM: Tumor/node/metastasis
WBRT: Whole-brain radiation therapy
